# Precise somatic genome editing for treatment of inborn errors of immunity

**DOI:** 10.3389/fimmu.2022.960348

**Published:** 2022-08-26

**Authors:** Qingzhou Meng, Haixiang Sun, Jianghuai Liu

**Affiliations:** ^1^Reproductive Medicine Center, The Affiliated Drum Tower Hospital of Nanjing University School of Medicine, Nanjing, China; ^2^State Key Laboratory of Pharmaceutical Biotechnology and MOE Key Laboratory of Model Animals for Disease Study, Model Animal Research Center at Medical School of Nanjing University, Nanjing, China

**Keywords:** inborn errors of immunity (IEI), hematopoietic stem cell (HSC), gene therapy, genome editing, base editing, prime editing

## Abstract

Rapid advances in high throughput sequencing have substantially expedited the identification and diagnosis of inborn errors of immunity (IEI). Correction of faulty genes in the hematopoietic stem cells can potentially provide cures for the majority of these monogenic immune disorders. Given the clinical efficacies of vector-based gene therapies already established for certain groups of IEI, the recently emerged genome editing technologies promise to bring safer and more versatile treatment options. Here, we review the latest development in genome editing technologies, focusing on the state-of-the-art tools with improved precision and safety profiles. We subsequently summarize the recent preclinical applications of genome editing tools in IEI models, and discuss the major challenges and future perspectives of such treatment modalities. Continued explorations of precise genome editing for IEI treatment shall move us closer toward curing these unfortunate rare diseases.

## Introduction

Recent developments in sequencing technologies have significantly accelerated the identification and determination of human monogenic immune defects. It has become evident that the collective prevalence of such genetic diseases is much higher than previously considered (at a scale of 1/1000 to 1/5000 births) (1). Traditionally known as “primary immunodeficiencies”, these defects are now commonly designated as inborn errors of immunity (IEI), a term better reflecting the presentation of a broad spectrum of immune-related phenotypes. Indeed, IEI patients can feature not only susceptibility to infections, but also autoimmunity/autoinflammation, allergy and malignancy (1–3). Additionally, even patients with the same mutations may manifest heterogeneous phenotypes. Active investigations of this expanding catalog of disorders have provided invaluable genetic and mechanistic insights on the development, activation and regulation of the human immune system, and its interactions with pathogens.

Such efforts have also brought advances to the treatment outlook for IEI. The Increased understandings to the molecular underpinning of various forms of IEIs have established the basis for some targeted therapeutic approaches, by which the defective components and pathways in immunity may be supplemented or pharmacologically targeted (4, 5). On the other hand, since a majority of IEI affecting the immune cells, hematopoietic stem cell transplantation (HSCT)-based therapies are considered to be curative for IEI (4, 6, 7). Nonetheless, allogeneic HSCT has long been limited by the need for finding the donors with optimal HLA genotypes, and by the potential risks of graft-versus-host-disease (GVHD) with severe consequence. Alternatively, the use of gene-corrected autologous hematopoietic stem cells (HSCs) removes such immunological barriers (7, 8). Indeed, viral vector-based gene therapies aimed at correcting the faulty genes in the autologous HSC compartment have made steady progress over the last 25 years (9). Particularly, the recent revolution in genome editing technologies has provided more advanced tools toward the promise of achieving precise and long-lasting management of IEIs (9, 10).

**Figure 1:**
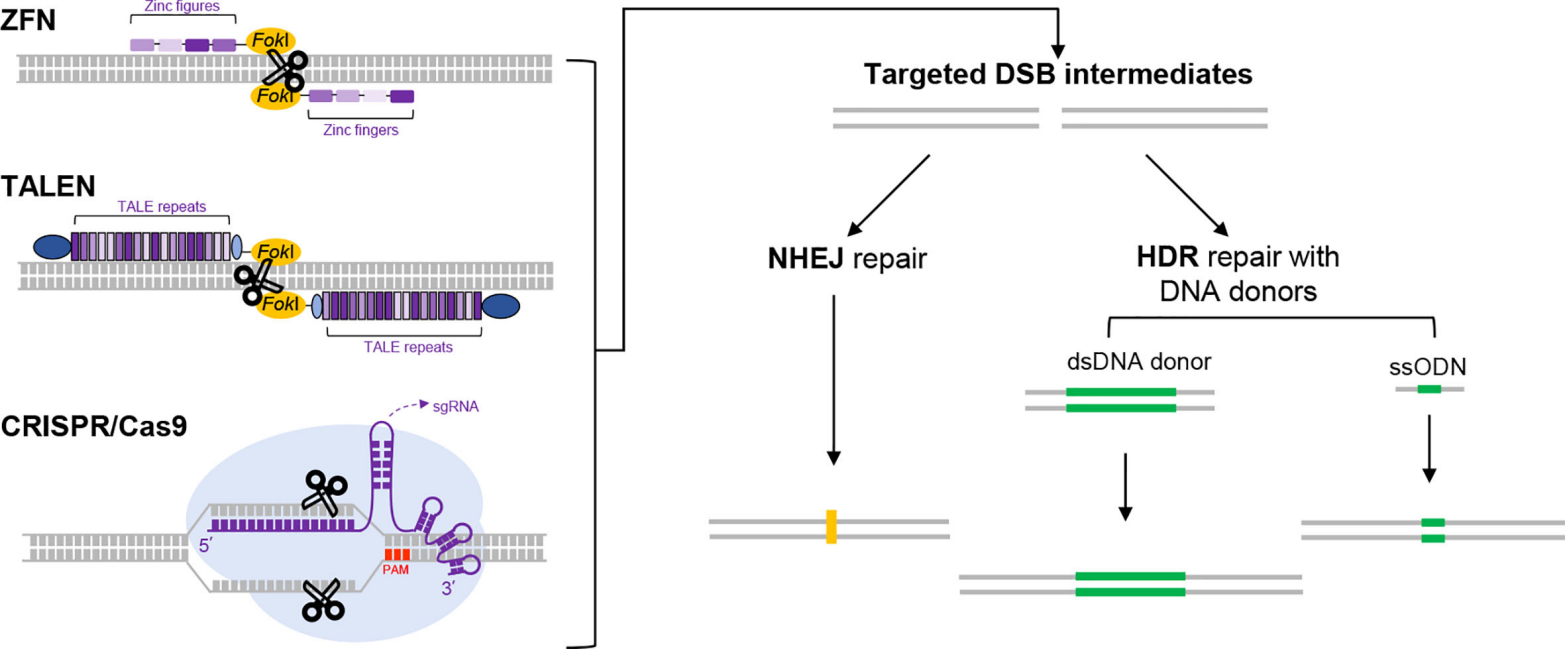
The use of programmable nucleases for genome editing. Three major programmable nucleases, i.e., ZFNs, TALENs and CRISPR/Cas9 are schematically illustrated on the left part. ZFNs and TALENs are respectively composed of sequence-specific DNA-binding protein modules linked to a nonspecific DNA cleavage domain (derived from *Fok*I endonuclease). In ZFNs, an array of zinc figures (small blocks in different shades of purple), each specifically recognizing a 3-bp of DNA sequence, are assembled in tandem to program a specific DNA-binding event. In TALENs, variants of TALE repeats correspond to each of the four single bases. Therefore, programmed DNA binding by TALEN is enabled by an assembled array of TALE repeats (dense purple bands). Two other constant domains in TALEN are also depicted in dark and light blue, respectively. For DNA cleavage to occur, the *Fok*I domain requires dimerization. As a result, only two adjacent targeting events by a pair of targeting ZFN or TALEN monomers in correct orientations would trigger double-strand break (DSB) at a specific locus. The mechanistic principle for CRISPR/Cas9 (a prototype for many CRISPR/Cas tools) is different from those for ZFNs and TALENs. It employs a Cas9 protein and an engineered guide RNA. The Cas9 protein features two nuclease domains, each responsible for cleaving one strand of DNA. The engineered guide RNA (in a form of single guide RNA, sgRNA [purple]) directs Cas9 to the DNA target by base-pairing, which results in generation of a targeted DSB. The target sequence for CRISPR/Cas9 also requires an immediately adjacent PAM sequence (shown in red). Regardless of the nuclease tools used, the DSBs generated would stimulate cellular DNA repair mechanisms (right part), in the form of the error-prone nonhomologous end joining (NHEJ) or homology-directed repair (HDR). The former pathway often leads to uncontrolled insertions or deletions of bases (indels, shown in yellow) around the break site, which tends to cause gene disruptions. The latter pathway engages precise repair directed by homologous donor DNA. In practice, when the cells are supplied with a donor DNA template (either as dsDNA or single strand oligodeoxynucleotides [ssODN] as indicated) containing the desired edits (green) and flanking homologous regions, the HDR pathway can lead to precise genome editing outcomes.

Genome editing refers to the technologies that enable programmed genetic modifications at specific locations in the genome (11). Fundamentally, these technologies depend on the ingenious design of different forms of programmable, sequence-specific nucleases (**Figure 1**). Targeted cleavage on the genome would drive DNA repair at the locus, which may subsequently lead to intended genetic changes (12). The first two major systems are the Zinc-finger nucleases (ZFN) and Transcription activator-like effector nucleases (TALEN) (13, 14). Therein, the Fokl nuclease is respectively fused to the assembled arrays of modular Zinc-fingers and Transcription activator-like motifs, which in turn mediate specific sequence recognition. Not long after the development of ZFN and TALEN, a third system originated from the bacterial defense mechanism exploded into the scene. This system is referred to as the Clustered Regularly Interspaced Short Palindromic Repeats/CRISPR-associated protein (CRISPR/Cas), corresponding to the nomenclature of the related genetic components in the prokaryotes (15). In general, the CRISPR/Cas platform is assembled with the Cas nuclease and a guide RNA component that contains a programmable sequence motif (a spacer of ~ 20-nt) complementary to the specific DNA target. Besides its potent targeting activity, this system also features unprecedented convenience and versatility in applications (16, 17). It is currently adopted as the most popular framework for constructing genome editing tools. Since its introduction in 2012, CRISPR/Cas technology has undergone extraordinary developments. As a result, these revolutionary tools have made strong impacts in all fields of life sciences.

**Figure 2:**
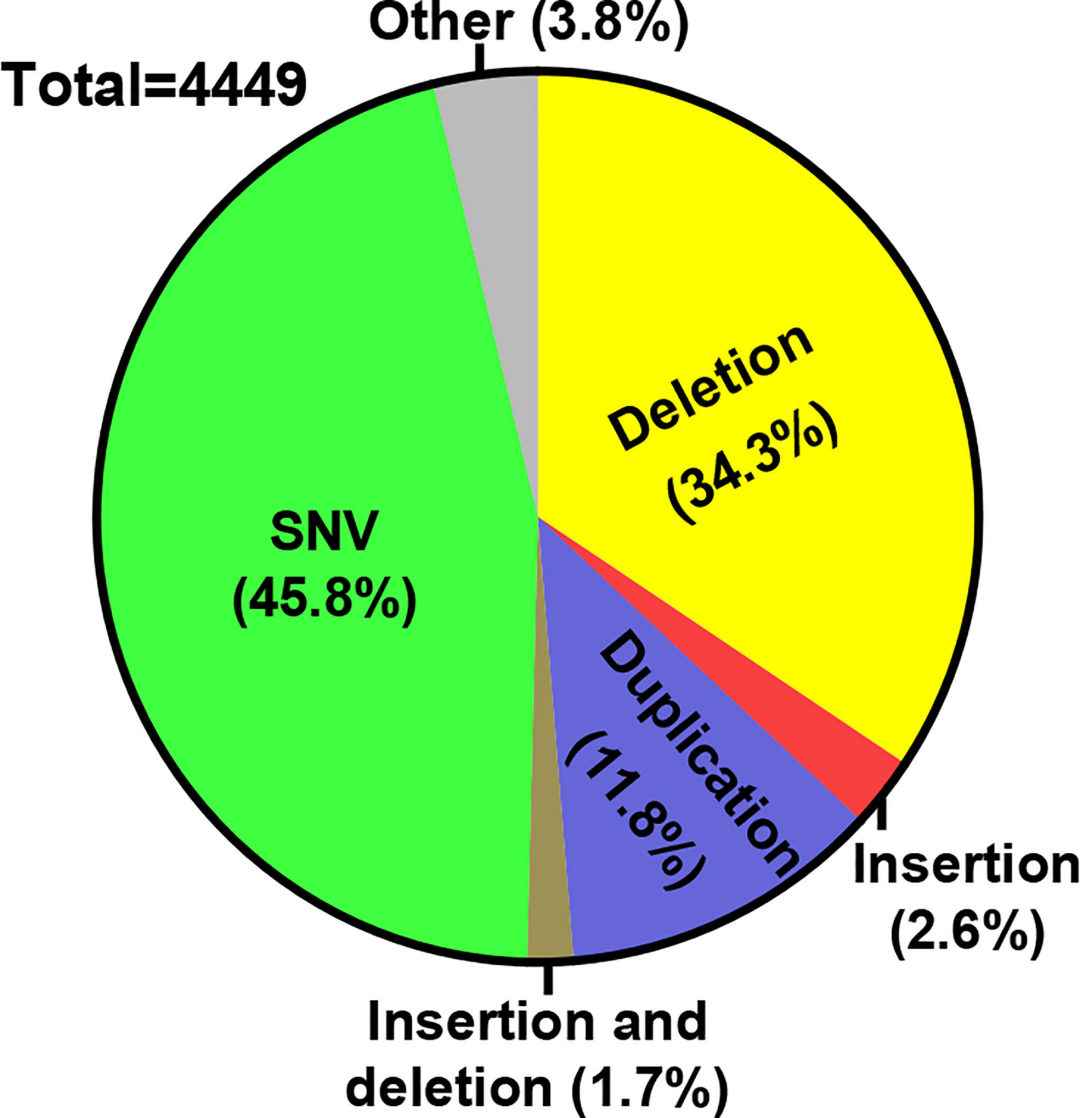
Distribution of IEI-associated variants. A survey of ClinVar database was conducted (June, 2022) for the distribution of the pathogenic variants associated with a keyword of “immunodeficiency”. The numbers of different variant types belonging to each of the indicated categories were filtered. Subsequently, the percentages for each category of variants within all different variants are marked on the pie graph.

The powerful advances in genome editing technology have reshaped the landscape for future treatments of genetic diseases. For IEIs, in particular, considering the clinical feasibility of using genetically corrected autologous HSCs to normalize immunity, it is anticipated that the genome editing-based strategy shall evolve into promising treatment options (9, 10). A brief survey of the ClinVar database (18), with the keyword of “immunodeficiency”, shows that the majority of the associated pathogenic variants belong to the categories of single nucleotide variants (SNV), deletions and/or insertions (**Figure 2**). Indeed, many of the available genome editing tools have shown the capacity to make (or reverse) these types of genetic changes (11, 19). Here, we will introduce the latest developments in CRISPR/Cas tools for precise genome editing. We will provide an overview for the latest preclinical applications of genome editing for IEI treatments. Furthermore, the challenges and future prospects for translating genome editing technology to the bedside of IEI patients will be discussed. Due to the focused theme of the present work and space limitations, we sincerely apologize to those authors whose valuable contributions to the field are not included in this review.

## The spectrum of IEIs and the underlying gene defects

The list of different forms of IEIs has greatly expanded over the last decade, thanks to the revolution in sequencing technologies. Based on the recent classification by International Union of Immunological Societies in 2019, IEI disorders encompass 430 distinct defects (1). According to their overall phenotypic features, these disorders can be divided into 10 broad categories: “combined immunodeficiencies”, “combined immunodeficiencies with syndromic features”, “predominantly antibody deficiencies”, “diseases of immune dysregulation”, “congenital defects of phagocytes”, “defects in intrinsic and innate immunity”, “autoinflammatory diseases”, “complement deficiencies”, “bone marrow failure”, and “phenocopies of IEI”. It is also clear from the accumulated knowledge regarding these genetic diseases, certain gene defects (or given alleles) may drive a spectrum of phenotypes, whereas similar phenotypes may also be attributed to defects in different genes (1, 3). Additionally, some of the defects would exhibit incomplete penetrance. The significantly improved understandings toward the genetic basis of IEI have not only opened up many options of targeted therapies, but also highlighted the potential of developing curative genetic therapies.

The majority of IEI defects primarily affect the immune cell compartments originated from HSCs (5, 7). Indeed, HSCT treatments have shown successes against the most severe forms of IEI that are refractory to other medical therapies, including severe combined immunodeficiencies (SCID), combined immunodeficiencies, chronic granulomatous diseases (CGD), Wiskott-Aldrich syndrome (WAS), and bone marrow failures (6, 7, 9). However, allogenic HSCT is limited by the difficulties of finding the optimal HLA-matched donors. When alternative donor strategies are used, the managements against conditioning morbidities, graft rejection and later GVHD present substantial challenges (7). On the other hand, the transplant of autologous, genetically engineered hematopoietic stem and progenitor cells (HSPCs) would avoid such immunological barriers. In principle, the defective HSPCs from the patients may be stably transduced *ex vivo via* a viral vector that produce the functional gene product, and subsequently are engrafted back to reconstitute (at least partially) the hematopoietic system. Over the past several decades, marked progresses have been made on the development of *ex vivo* manipulation of HSCs *via* viral gene vectors, and the subsequent autologous HSCT in clinical settings (8, 9, 20). Currently, the use of self-inactivating lentiviral vectors has shown good promise as a safe and effective platform for HSC gene therapy, as demonstrated in clinical trials for X-linked SCID, adenosine deaminase (ADA)-deficient SCID, Fanconi anemia (FA), CGD and WAS (21–25). Moreover, similar gene therapy approaches for other IEIs including RAG1-deficient SCID and Artemis -deficient SCID have also entered clinical stages (see NCT04797260 and NCT03538899), based on favorable outcomes from preclinical studies (26, 27). However, when implementing the current generation of HSC gene therapy tools, the permanent, semi-random integration of lentiviral vectors in the genome may still pose long-term risks. For genes whose expressions requires cell type- and/or activation status-dependent regulation (such as *CD40L*, mutated in X-linked hyper-IgM syndrome), the viral vector-dependent, non-physiological transgene expression can cause safety concerns (28), or conversely, may lack the required robustness upon activation (29). In addition, such “gene addition” approach is not suitable for correcting gain-of-function mutations, and may not be effective in rescuing certain dominant-negative defects (9). In this regard, precise mutation-targeted, “gene correction” methods are believed to represent the future tools-of-choice for gene-based therapy of IEIs.

## Genome editing

Effective modification of the genomes in animal cells had been a challenging task until recently. The emergence of genome editing technologies in the late 2000s and early 2010s indeed marked the beginning of a new era (16, 17). Empowered by these ground-breaking technologies, making genetic changes in a cell has become quite accessible. The tremendous development of the genome editing toolbox over the last decade has also brought the hope that the ultimate cures for many genetic diseases, through somatic genome editing, are on the horizon.

### CRISPR/Cas9 genome editing

The first generation of genome editing technologies are based on the engineered nucleases that can program a double stranded DNA break (DSB) at a site(s) of interest. The popular classes of programmable nucleases include ZFNs (30), TALENs (31, 32) and CRISPR/Cas9 (15, 33, 34), which emerged successively. Among these, the CRISPR-Cas9 has become the most widely used platform owing to its potent activity, unprecedented convenience and versatility. Originally identified as a bacterial adaptive defense system against phages, the CRISPR/Cas9 can specifically cleavage foreign DNA sequences based on the targeting information contained in a guide RNA moiety (formed with a tracrRNA and a crRNA). In the commonly used platform for genome editing, the *Streptococcus pyogenes* Cas9 protein (SpCas9) is directed by an engineered guide RNA (single guide RNA, sgRNA) to make DSB cut at the target DNA sequence (See **Figure 1**) (16, 17). Each sgRNA is simply customized by a stretch of a 20-nt spacer sequence that aligns with a target DNA sequence. One restraint for the target DNA sequence is its immediate adjacency to a downstream protospacer adjacent motif (PAM). For SpCas9, the classical PAM sequence is 5′-NGG-3′, which is found very frequently in a genome space. Other natural CRISPR/Cas systems (e.g., SaCas9 or Cas12a) operating in similar or slightly deviant manners have also been later harnessed for genome editing (35). Importantly, with their recognition of corresponding PAM motifs, these different tools further expand the targeting scope for CRISPR/Cas-based genome editing.

The nuclease-generated DSBs activate the cellular repair pathways (see **Figure 1**), which can result in non-homologous end-joining (NHEJ) (13, 33, 34, 36) or the template-dependent, homology-directed repair (HDR) (12, 33, 34, 37, 38). The first pathway operates actively in most cell types, which often drives formation of indels at the targeted sites. These indels are sometimes sufficient to cause loss-of-function in genes or regulatory elements (39–41). The use of paired sgRNAs can lead to more definitive knockouts (42). In the presence of a co-introduced homologous template, the cells may also choose the second repair option, which can lead to precise installation of designed sequence changes (33, 34, 38). Nevertheless, establishment of such HDR-dependent gene knock-in often requires screening or selection, owing to the relatively lower efficiency of HDR repair (43). Further technical advancements to enhance the preference of repair *via* HDR vs NHEJ represents an intensive area of research (19).

A major safety concern for genome editing is the off-target effects by the programmed nucleases. For instance, Cas9 may cause cleavage at certain off-target sites bearing high similarity to the sequence of sgRNA spacer (44). Studies have shown that minor mismatch, especially at the PAM-distal positions, can sometimes be tolerated (44, 45). In general, limiting the amount of the Cas9/sgRNA complex and/or preventing its prolonged presence can effectively reduce off-target cleavage (42). Other developments include the engineering of high-fidelity Cas9 (46–49), and the use of modified sgRNA architectures (50, 51) to lower the chances of Cas9 cleavage at imperfectly matched sites. Since the efficiency and accuracy for a given CRISPR/Cas9 platform tend to inversely correlate (52), to further seek strategies for reducing off-target cleavage while maintaining the on-target genome editing efficiencies remains an important goal.

Although Cas9 cleavage-dependent genome editing approaches have been widely applied in cells and model organisms, the generation of DSB intermediates poses concerns for applications that require greater levels of safety, such as therapeutic genome editing. Indeed, studies have reported that Cas9-generated DSBs may cause certain levels of large deletions, genomic rearrangements, and even potentially chromothripsis in different cell types including human HSCs (53, 54). The potential of off-target DSBs exacerbates such risks. Moreover, although the introduction of a repair template can lead to engagement of the HDR pathway, the co-existing NHEJ pathway often complicate the repair outcomes (11). To address these issues, a number of innovative, precise genome editing platforms that avoid the requirement of DSB formation have been established.

### The base editing platform

**Figure 3:**
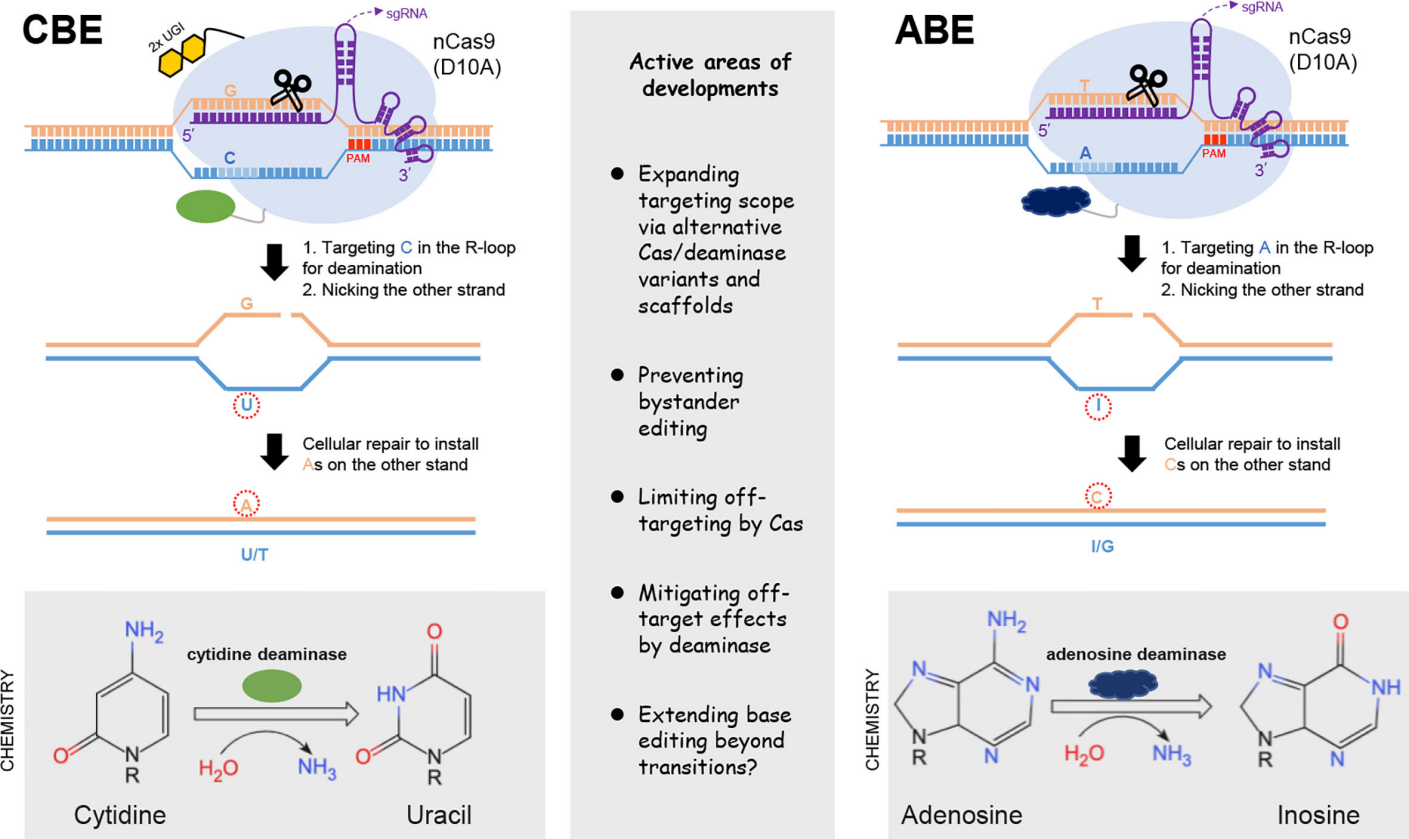
Base editing tools. The two major classes of base editors are illustrated on the left and right parts, respectively. CBE is constructed by fusing the Cas9 nickase (nCas9) with a cytidine deaminase enzyme, which targets the Cs within the exposed R-loop for conversion into U, the latter serving as a temporary surrogate for thymidine in base pairing properties. The uracil glycosylase inhibitor domains (2xUGI, in yellow) engineered to prevent base excision of the U are also shown. The activity window of the initially developed CBE often covers positions 4-8 (schematically indicated with light blue). The adoption of nCas9 in CBE is by design. Its activity would nick the unedited strand, thereby signaling the cellular repair to install the corresponding As on the complementary strand. The design of ABE follows similar principles, except for the employment of an evolved, DNA-targeting adenosine deaminase. Deamination of adenosine produces inosine, which serves as a temporary surrogate for guanosine. Note that no inhibitory domains against base excision of I are required for optimal ABE activities. Some key considerations for the current developments of base editors are summarized in the middle part.

The emergence of base editors (BE) underscores the power of harnessing DNA base-modification activities for precise genetic manipulation. The common base editors can be divided into cytosine- or adenine-base editors (CBE and ABE), which respectively induces C-to-T and A-to-G base transitions in the target sequences (55–57). These base editors also mostly adopt the CRISPR/Cas9 framework for target binding, by the use of Cas9 form deficient in dsDNA cleavage activity (**Figure 3**). In principle, CBE is constructed by fusing the mutant Cas9 first with a cytidine deaminase enzyme, which recognizes the Cs within the mutant Cas9/sgRNA-exposed stretch of single-stranded DNA (ssDNA) as substrates (55). This subsequently leads to conversion of cytidine to uridine, the latter serving as a temporary surrogate for thymidine in base pairing properties. Furthermore, to inhibit the base excision repair for uracil removal, the CBE is also engineered to include an additional uracil glycosylase inhibitor domain. Thirdly, the deliberate use of a Cas9 nickase (nCas9 [D10A]), capable of nicking the unmodified single DNA strand, can direct the cellular repair pathways to “re-write” the sequence near the nick. This would eventually lead to a permanent change of C·G into a T·A base pair (**Figure 3**).

The blueprint for the initial establishment of an ABE is similar as above, except for the employment of a specific deaminase activity to first convert an A to an inosine, the latter serving as a temporary surrogate for G. Since there is no natural enzyme that catalyze this reaction on a DNA strand, a directed evolution approach was applied toward an *E. Coli* tRNA adenosine deaminase (ecTadA). This led to the establishment of an engineered TadA that can catalyze adenosine deamination in the target sequence when fused to the Cas9 moiety (nCas9) (56). Interestingly, no requirement for an additional domain to inhibit base excision at inosine is required for activity enhancement of the ABE. Similar as above, the use of nCas9 in ABE would also shift the strand preference for the repair pathway to promote the eventual change of an A·T into a G·C pair (**Figure 3**). Collectively, the abilities the cytosine and adenine BE to mediate targeted base transitions have strong practical implications. Indeed, more than half of currently cataloged pathogenic SNP in the human genomes are base transitions in reference to the WT alleles (57). It is also important to note that, the initially described CBE and ABE tools already showed potent editing activities. Given their proper complementation to the DSB-dependent editing tools, the BEs platforms have undergone active development since their introductions.

The first nCas9-based CBE and ABE respectively showed activities against Cs and As situated in a particular window (approximately at position 4-8, when the corresponding PAM motif is counted as position 21-23) of the target sequence (57). For more flexibility with the target scope, many other base editors have been developed based on different Cas variants (19, 58–60). These BEs show different PAM requirements, and feature their respective editing windows. As more Cas domains with less restricted PAMs are becoming available (61), increasing proportions of C and A base positions (and by extension their complementary Gs and Ts) in the genome become targetable. It is estimated that, by the use of different Cas variants, the expanded targeting scope of the BEs are sufficient to cover most pathological base transitions cataloged in the ClinVar database (18, 19). Moreover, the choice of different natural/evolved deaminase domains (62–64) and other modifications on BE architectures (65, 66) can influence not only the BEs’ editing window features, but also their substrate context preferences (55, 67). Practically, while BEs with wider editing windows are more likely to cover a particular base target, they are linked with a higher risk of generating “bystander” mutations at unintended Cs or As within the targeting window. When the bystander base changes are detrimental and need to be avoided, BEs with narrower editing windows or requiring a more restricted sequence context may be employed (58, 68).

Off-target effects by BEs can be attributed to Cas9-dependent and Cas9-independent mechanisms. The base editors are recruited by the sgRNA to imperfectly aligned sites, potentially leading to deamination of Cs or As within the activity window. Since a subsequent nCas9-dependent strand nicking would significantly facilitate productive installation of base transitions at such unintended sites, the various strategies to mitigate Cas9’s off-target cleavage activities would also be effective in improving BE specificities (68–70). On the other hand, the Cas9-independent off-target base editing is believed to mainly result from the action of the deaminases on the transiently formed single-stranded DNA in the cells (71, 72). Such undesired effects can be substantially reduced by the adoption of certain mutant deaminase domains or by changes in BE architecture to install a stricter requirement of Cas9-mediated target binding for the deaminase actions (71, 72). In addition, the variously observed activities by BEs to edit cellular RNAs can also be mitigated by the use of further optimized deaminase domains (73, 74).

It needs to be noted that the current BE strategies are apparently not suited for making precise base transversions (a purine to a pyrimidine, or vice versa). Additionally, there is often a great need for installing other types of genetic modifications in research models and/or for translational applications. The prime editing (PE) platform are subsequently established to potentially fill such a gap.

### Prime editing

**Figure 4:**
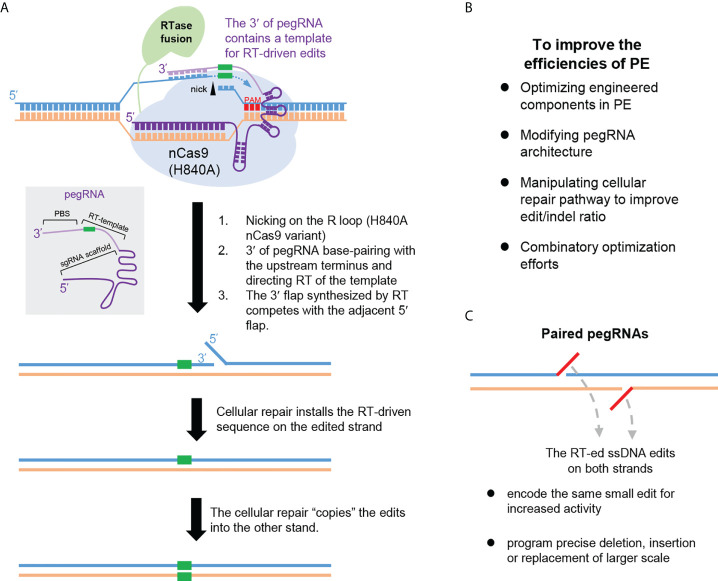
Prime editing. **(A)** The principle underlying the prime editor (PE) is illustrated. PE employs nCas9 (H840A) fused with a reverse transcriptase (RT) domain. PE is directed by pegRNA whose structure is schematically shown in a grey box. The pegRNA is composed of a targeting sgRNA scaffold and an extended 3′ region. This region can base pair with the nicked R-loop (via its terminal portion named primer binding sequence, PBS), and directs the ensuing reverse transcription (via its edits-containing segment named RT-template). The potential mechanistic stages underlying PE are depicted in order. **(B)** Major focuses for improving the PE efficiencies are summarized. **(C)** The advantages of PE with paired pegRNAs are summarized. Note that each pegRNA would enable reverse transcription of a segment of ssDNA (indicated in red). Programming the paired pegRNA sequences would enable increased PE activities, or empower precise editing of larger scales.

The recently emerged PE technology enables installation of various types of point mutations and small insertions/deletions (75), which represents a significant breakthrough in the field (**Figure 4A**). The basic PE system is composed of a fusion protein of nCas9 (H840A) and a reverse transcriptase (RTase) domain, together with an engineered prime editing guide RNA (pegRNA). The pegRNA consists of a targeting sgRNA scaffold and an extended 3′ region. Particularly, this 3′ region features a primer-binding site (PBS), and an adjacent sequence containing the intended edits. The latter sequence would serve as a template for reverse transcription (RT) and is named RT template. Guided by the pegRNA, the nCas9 (H840A) makes a nick on the sgRNA spacer-displaced ssDNA. By design, the PBS segment in the 3′ portion of the pegRNA forms base pairs with the sequence upstream of the nick. The adjacent RT template can then readily direct reverse transcription by the nCas9-fused RTase, which employs the nick-created 3′-end as the “primer”. Next, due to homology, the newly reverse-transcribed ssDNA may displace the nick-downstream DNA from base-pairing with the unedited strand. Following removal of such 5′-flap structure by cellular nucleases, nick ligation concludes the editing on the first strand. Further DNA repair to resolve the one side-edited heteroduplex can lead to permanent installation of the edits (**Figure 4A**). In aggregate, this forms an applicable platform named PE2. A subsequent development of the system (i.e., PE3) is by inclusion of a second sgRNA for nicking the unedited strand, to bias the repair pathway toward productive incorporation of edits into this strand (75).

Due to the adoption of a templated, reverse transcription-based mechanism for editing, PE can support a broad scope of precise genetic modifications with high purity (avoiding bystander mutations) (75). Importantly, likely owing to the involvement of multiple hybridization events, the PE platform also feature very low genome-wide off-target effects (75–77). Nevertheless, applications of PE in various systems have revealed a general trend of its suboptimal and inconsistent efficiencies. This has stimulated an active line of investigations to enhance PE efficiencies (**Figure 4B**). Against various potential bottlenecks for PE actions, several optimization strategies have been undertaken. These include the strategies to improve the chromatin accessibility of the targets (78), to optimize the reverse transcriptase domain (79), to modify the pegRNA architectures for increased levels/activities (79–82), and to manipulate DNA repair mechanisms to facilitate productive incorporation of the edits (83, 84). Combination of more than one strategy may enable further enhancement effects (83).

Other major developments in PE include to harness the use of paired pegRNAs (**Figure 4C**) for either activity enhancement (85, 86), or for inducing precise sequence deletion or replacements (87–89). In the former case, dual pegRNAs encoding the same edits on opposing strands would be used, so that precise editing on both strands may be engaged (85, 86). A similar strategy can be adopted for the induction of precise deletions, *via* assembling sequences on either side of the deletion as respective RT templates in the paired pegRNAs (88). This would support much longer (in kb-scale) deletions in comparison to the technological limit allowed by the use of a single pegRNA. Furthermore, by the design of complementary new sequences in the RT templates of the paired pegRNAs, precise insertion of a new sequence (in the scale of hundred-bp) in place of the original DNA segment between the nicks can be also achieved (87, 89). For instance, Bxb1 recombinase-recognized *attB* or *attP* sites can be precisely installed in the genome. When applied in conjunction with the site-specific recombinase technology, this can drive precise insertion of large DNA fragments (in kb-scale) (89). One other useful PE-related tool is the WT Cas9-based variant of PE (PE-nuclease, PEn) (90–92). Compared to the original PE, the PEn generates a DSB intermediate that significantly promoted the rates of installing RT template-encoded edits. However, the parallelly induced imprecise edits from the error-prone NHEJ represent an apparent limitation that need to be further addressed. Collectively, the high versatility of precise genome editing brought by the PE technology holds great promise for future therapeutic applications.

## Genome editing of HSPCs in IEI-related preclinical applications

The advances in genome editing technologies have suggested a promising direction for future treatments of IEIs. Similar to viral vector-based gene therapies, genome editing for correcting the mutant gene may be feasibly applied to the autologous HSPCs *ex vivo*, followed by transplantation of the treated cells back to the patients (8–10, 93). In this relatively new area of research, current investigations have focused on testing different genome-editing strategies in IEI-related preclinical models.

### Genome editing in SCID-X1

The mutations of the X-chromosome-located *IL2RG* gene are responsible for SCID-X1 (6). The resultant deficiencies in the common γ-chain-dependent cytokine signaling cause a complete block in the development of T and NK cells, in conjunction with functional defects in B cells. As substantial development had been made on SCID-X1 gene therapy, *IL2RG* genome editing in human HSPCs were carried out as proof-of-principle tests. A common correction strategy was adopted by various studies (94). Functional rescues of most pathogenic *IL2RG* mutations would be achieved by the targeted insertion of a WT *IL2RG* cDNA to the endogenous *IL2RG* locus *via* DSB-induced HDR. It was found that optimization of culture conditions to promote the *ex vivo* proliferation HSC was important, due to the low HDR activities in the normally slow-cycling HSCs (95). Initially, with the implementation of customized ZFNs (as mRNA) and a repair template in the form of an integrase-defective lentiviral vector, low but detectable levels of HDR-driven correction of *IL2RG* were achieved. Significant enhancements were later made by the use of an adenovirus-associated virus vector (i.e., AAV6)-based repair template (96, 97). By the use of CRISPR/Cas9 for DSB induction, together with the delivery of an AAV6 template, up to 45% of treated CD34^+^ HSPCs showed desired corrections (97). The *in vivo* multi-lineage repopulation by the gene-corrected cells was evidenced in the permissive, recipient mice. Collectively, these works have established an initial framework for the possibility of editing genetically deficient HSPCs for treatment of SCID-X1 and other IEIs.

### Genome editing in models of IEIs more challenging for gene therapy

The mutations affecting each of the 5 subunits of the NADPH oxidase system are responsible for CGD (6). Due to the role of NADPH oxidase in the generation of microbicidal reactive oxygen species in myeloid cells, patients are susceptible to recurrent severe infections, which reduces quality of life and cause mortality (98). The CGD is a challenging condition for gene therapy. A high rate of gene delivery to HSCs and an optimized level of engraftment are required, as the functionally restored myeloid cells do not show selective advantage *in vivo* (6, 10). Furthermore, the control of specific transgene expression in myeloid cells is desirable to limit ectopic transgene-induced toxicity to cells in other lineages (99).

The mutant *CYBB* gene (gp91 subunit) contributes to X-linked form of CGD (X-CGD). Previously, a lentiviral vector with a myeloid-specific chimeric promoter has been developed for gene rescue in X-CGD (99, 100). Recently, a clinical trial on such a X-CGD gene therapy platform has shown promising efficacy in 12 months of follow-up (21). Nevertheless, a gene correction strategy at the endogenous locus would more closely mimic physiological expression of the corrected genes, potentially enhancing the treatments’ efficacy/toxicity profiles. An earlier study using ZFN and an AAV6-based template for knocking-in *CYBB* gene to a genomic “safe harbor”, i.e., the AAVS1 locus, in human HSPCs paved the way for future developing gene-correction at the endogenous locus (101). In later investigations for correcting a point mutation in exon 7, CRISPR/Cas9 was used in conjunction with an ssDNA oligonucleotide repair template to achieve moderate levels of gene correction (102). By further promoting cellular HDR pathway *via* inhibition of 53BP1 activity, restoration of protein expression was achieved in high percentages (~ 65%) of myeloid cells derived either from *in vitro* edited HSPC, or from long-termed engrafted human BM cells (103). Such substantial correction rates by genome editing suggests its potential in future treatment of X-CGD. It needs to be noted that as *CYBB* is affected by a large number of pathogenic mutations (104), the feasibility for developing such *in situ* correction approaches into potential X-CGD treatment modalities would require future demonstration.

The autosomal forms of CGD include those caused by mutations affecting the p47 subunit of NADPH oxidase (p47-CGD) (105). Interestingly, one specific homozygous 2-nt deletion (ΔGT) in exon 2 the *NCF1* genes (encoding p47) represents the predominant defect in p47-CGD, which indeed accounts for ~ 20% of all CGD cases. As two pseudogenes for *NCF1*, i.e., *NCF1A* and *NCF1C* harbor the same deletion, the patient-related ΔGT in *NCF1* is likely to be caused by the pseudogene-related gene conversion events (106, 107). By adopting a system of ZFN coupled with an AAV6-based template, some *in vitro* myeloid progenies of edited HSPCs showed apparent rescue of p47 function. The work further provided evidence that functional p47 rescue may have arisen from the correction of the pseudogenes (108). Further tests for *in vivo*, long-term correction of p47 function are still warranted. Nevertheless, due to the relative prevalence of this specific ΔGT mutation in p47-CGD, further development of such an *in situ* correction strategy would have therapeutic implications.

Another group of intensively studied IEI is WAS. The X-linked WAS disease is caused by mutations in the *WAS* gene (encoding WASp), a regulator of actin polymerization with broad pattern of expression in hematopoietic cells (109). The deficiency of WASp function leads to dysfunctions in all mature hematopoietic cells, except the red blood cells. In accordance, WAS patients suffer from severe platelet defects and complex immunodeficiency (110). Clinical trials have been undertaken to assess lentiviral vector-based gene therapy for WAS (23, 111, 112). To ensure safety, a 1.6-kb endogenous WAS promoter was used in the self-inactivating lentiviral vector to control WAS expression. The results showed sustained clinical benefits for treated patients (23). Nevertheless, although the therapeutic vector largely corrected the dysregulated T lymphocyte compartment, the function of the platelet was only partially restored (23). One likely reason for such incomplete rescue is that the physiological WAS expression is not fully recapitulated under the control of the 1.6-kb promoter. Therefore, a recent study was carried out to insert a WT *WAS* cDNA to the endogenous *WAS* locus in human HSPCs *via* CRISPR/Cas9 and an AAV6-based template (113). Interestingly, this led to more efficient restoration of WASp expression, indicated by the positive percentage (~ 50%) and by per cell level (equivalent to WT level), as compared to the parallelly tested lentiviral transduction system (described above). Moreover, macrophages, platelets and T lymphocytes derived from the edited HSPCs *in vitro* showed restored functionality. The long-term (up to 26 weeks) multi-lineage repopulating ability (~ 37%) by the edited HSPCs were further established in transplanted mice (113). Overall, this high-performance gene-correction platform for WAS warrants further development, as HDR-mediated *in situ* cDNA insertion would enable functional restoration of WASp activity affected by many different mutations (> 300).

### Genome editing by HDR in other IEI models

X-linked hyper-IgM syndrome (XHIM) is caused by mutations in the *CD40LG* gene encoding CD40 ligand (CD40L) (114). Activation of T cells lead to induced cell-surface expression of CD40L, which in turn acts *via* interaction with CD40 on the B cells to drive class-switch recombination at the immunoglobulin heavy-chain gene (115). Due to the lack of IgG/A/E antibodies, the XHIM patients are highly susceptible to infection and autoimmunity, and exhibit poor long-term prognosis (116). Lessons from earlier development of CD40L gene therapy models have underscored the unmet need for proper transcriptional control of CD40L, as ectopic expression of this transgene could cause abnormal lymphoproliferation (117). Recently, targeted insertion of a WT *CD40LG* cDNA to the endogenous locus in human HSPCs has been tested *via* the use of programmed nucleases and an AAV6-based template. The results showed reasonable rates of gene integration. Normal multilineage differentiation of the edited cells *in vitro* and *in vivo* was also demonstrated (118).

Another example of X-linked IEI is the IPEX (Immune dysregulation, polyendocrinopathy, enteropathy, X-linked) (119). This genetic autoimmune condition is caused by the mutations in the *FOXP3* gene, which encodes an essential transcription factor for the maintenance and function the regulatory T cells (Treg). The effector T cells (Teff) also transiently up-regulate FOXP3 expression following TCR activation (120). Therefore, deficiency of FOXP3 profoundly impair the function of both Treg and Teff cells, driving severe autoimmune manifestations (121). The tight cell type- and activation status-dependent FOXP3 expression patterns has hampered development of viral vector-based HSPCs gene therapy for IPEX (122). Aimed at enabling physiological correction of *FOXP3*, a recent study tested the strategy of integrating *FOXP3* cDNA to the endogenous locus, *via* genome editing with CRISPR/Cas9 and an AAV6 template. This led to functional restoration of Treg and Teff cells from IPEX patients. Additionally, FOXP3-edited HSPCs showed long-term engraftments and maintained multi-lineage differentiation potential in the recipient mice (123).

In another combined immunodeficiency disease, the X-linked MAGT1 deficiency with increased susceptibility to Epstein-Barr virus and N-linked glycosylation defect (XMEN), the mutated gene encodes an Mg^2+^ transporter and a non-catalytic subunit of an oligosaccharyltransferase (124). In addition to Mg^2+^ abnormalities, the deficient N-glycosylation of certain cellular proteins such as the NKG2D receptor on the CD8 and NK cells cause immune defects and the marked susceptibility to chronic EBV infections (125). Currently, effective therapeutic options for XMEN have not been developed. A recent work attempted to insert a WT *MAGT1* cDNA to the endogenous locus in human HSPCs *via* CRISPR/Cas9 and an AAV6-based template (126). To enhance the efficiency/toxicity profile of precise editing in HSPC, combined inhibition of NHEJ pathway and p53-mediated damage response was applied. This led to efficient correction of MAGT1 in patient-derived HSPCs, and restoration of T and NK cell functions (126). The edited HSPCs also showed long-term engraftment to support multi-lineage development in transplanted mice. In addition, efficient direct correction of patient-derived T cells was demonstrated with the same targeting strategy, which suggested a potential, shorter-term T cell-centered treatment strategy.

### Genome editing by NHEJ in Fanconi anemia

FA represents the most common inherited bone marrow failure syndrome, and is caused by mutations in any of the > 20 genes in the FA DNA repair pathway (127). As the hematopoietic compartment is particularly sensitive to deficiency in this pathway, the FA patients show progressive exhaustion of bone marrow reserve early in life (128). Notably, mutations in *FANCA* gene account for more than 60% of total FA, which defines the subtype of FA-A (129). Lentiviral vector-based gene therapy for FA-A are under clinical investigations (25, 130). WT *FANCA* gene addition *via* genome editing has also been reported in patient-derived HSPCs (131). However, the commonly used DSB/HDR genome editing strategy is conceivably suboptimal in this particular context, due to the impaired HDR in cells with deficiencies of the FA pathway (132, 133). On the other hand, a recent study explored an alternative strategy of harnessing NHEJ to rescue the coding frame of the mutant FA gene, by programming a DSB near the original mutation (134). The relatively enhanced NHEJ activities (over HDR) in the FA HSC cells are also conducive to the implementation of this strategy. Importantly, the functionally corrected cells would exhibit selective advantages over the uncorrected or mis-corrected cells. Indeed, the authors showed that despite the imprecision in initial editing outcomes, cells with the therapeutic alleles at *FANCA* increased progressively *in vitro* and *in vivo* (134). Although the long-term safety and therapeutic effects in this model await to be investigated, these results suggest a convenient and economic approach for FA gene corrections, where a relatively low number of initially corrected HSPCs may become favorably selected to provide phenotypic rescue over time. Whether such a strategy may be suitable for other types of IEI conditions are exciting avenues for future research.

## Challenges and outlooks for the development of genome editing-based IEI treatments

The remarkable developments in genome-editing technologies have opened up new research and therapeutic opportunities for IEIs. It is now generally convenient to establish precisely genome-edited animal or cell models carrying the patient-specific mutations (16). Such models provide invaluable tools for systematic exploration of mechanistic links between IEI genotypes and phenotypes, which shall subsequently suggest therapeutic strategies in a disease-specific manner. Importantly, as genome editing is moving into clinical applications for other blood disorders, i.e., β-thalassemia and sickle cell disease (135, 136), we have herein placed our main focus on the therapeutic aspect of genome editing in IEIs.

### A framework based on experiences from conventional gene therapies

Despite the high burden for implementation, the vector-based gene therapies for some IEIs have currently reached clinical stages (9). Further exploration of genome-edited HSPC therapies should be driven by previous experiences from the conventional gene therapies. Based on current technical feasibility, *ex vivo* gene correction followed by *in vivo* transplantation shall remain the major treatment format (8).

In principle, various genome editing tools developed so far can be applied to drive targeted, somatic corrections of genetic defects underlying IEIs. Such a strategy may potentially exhibit advantages in safety, efficacy and application scope, compared to the current viral vector-based gene therapies (9, 10). Nevertheless, there are still many challenges that need to be carefully addressed, before these new technologies can be translated into valid IEI treatment options. It is important to note that a number of important considerations regarding HSPC collection, *ex vivo* HSPC culture, optimal patient conditioning regimens, and transplanted HSPC have been thoroughly discussed recently by some excellent gene therapy-related reviews (6, 9, 20). Therefore, only considerations that are specific to the genome editing approaches would be briefly covered below.

### Challenges and outlooks for CRISPR/Cas9-based therapeutic strategies

Despite a large list of available genome-editing tools (11), most of them are yet to become applicable for the clinics. For genome editing to develop into a common treatment option against the highly diverse IEI-causing mutations, its universal safety, adaptability, and effectiveness would need to be firmly established. Currently, the CRISPR/Cas9-based genome editing platform has undergone the most extensive and advanced developments (19). Many studies have explored the DSB/HDR mechanism for cDNA knock-in at the endogenous locus, potentially representing an “one-size-fits-all” type of correction strategy for a given gene mutated in IEI patients. One of the most efficient delivery routes for genome editing of HSPC is *via* electroporation of Cas9 machinery in the form of a ribonucleoprotein (RNP) complex (8, 93), where the sgRNAs may be chemically synthesized and modified for higher activities (137). To promote HDR knock-in while limiting the DNA template-triggered toxicity, the co-delivery of AAV6-based repair template is commonly adopted (95). However, it is often challenging to consistently achieve high rates of HDR in HSPCs, especially in the primitive HSC compartments, which would cause difficulties for long-term engraftment of corrected cells. Studies on the culture conditions or molecular manipulations to increase the proliferation/expansion of HSCs, and to promote their HDR activities continue to constitute an active area of research (93, 138). Additionally, clonal tracking investigations need to be carried out more extensively, so that protocols leading to optimized clonal repertoire of the gene-edited HSPCs can be established (139).

The off-target effects by Cas9 often raise concerns regarding unintended, detrimental modifications to the genomes. Considerable developments have been made on improving the fidelity of Cas9 (19). However, further investigations on the off-target events based on sensitive and unbiased detections in clinically relevant models, as well as on the long-term safety profiles of edited cells are still highly warranted (9, 11). Additionally, due to the possibility of DSB intermediates to induce large genomic abnormalities, careful testing and optimization for the current DSB/HDR strategy for gene correction in HSPCs still represent a priority issue (11). Furthermore, despite the fact that cDNAs placed under the endogenous promoters are under similar transcriptional control as the endogenous gene, this may not be sufficient to recapitulate the overall regulation of the gene, due to the likely omission of other regulatory elements such as the intronic elements (10). Genes with multiple functional splicing variants are also unlikely to be fully rescued by a single form of cDNA.

On the other hand, since most of the current preclinical investigations have focused on the Cas9-dependent knock-in strategies, they are further developed regarding feasibility and reliability. Given further improvements in the efficacy/toxicity profiles and reductions in practical costs associated with *ex vivo* editing, we anticipate that certain editing regimens may be translated to clinical stage in the near future.

### Challenges and outlooks for BE- or PE-based therapeutic strategies

In contrast to the Cas9-dependent genome-editing approach, the later developed base editors and prime editors provide opportunities for precise, *in situ* modification of the gene targets, without the requirement of DSB and introduction of a DNA template (19, 57). The fact that both BE and PE do not rely on HDR (inefficient in primitive HSCs) for installation of mutations also present important advantages for their potential application in HSPC therapy. Although yet to reach clinical stages for any indications, these newer tools already show strong potential for future therapeutic applications.

However, gene corrections by these approaches need to be designed on *per* mutation basis. This is currently a demanding task, considering that these editing platforms are still under extensive developments regarding their editing safety, on-target efficiency and purity profiles. The relative bulkiness of base editors and prime editors may impair their deliveries to the cells (11). Moreover, the relatively large sizes of pegRNAs and their activity-enhanced variants also become hurdles for PE applications that require chemically synthesized guide RNAs (81).

Most current base editors can only program base transitions, but not base transversions. Furthermore, it remains challenging to target a particular position without potentially engaging bystander editing. A possible solution against such editing impurities is by testing and selecting from base editors with reportedly different PAM restrictions, activity windows and context preferences (19). However, for such a strategy to become applicable, systematic and global characterizations and benchmarking of many different base editors would be required.

Compared to the base editors, the editing scope by the prime editors are apparently broader (75), which shall cover the majority of the pathogenic alleles in IEIs. However, their overall suboptimal and inconsistent activities still represent a major limiting factor. Besides further improving the architecture of PE tools, continued global-scale targeting experiments are required to further refine the rules governing the effectiveness by pegRNAs (140).

Despite the challenges, we anticipate that the BEs and PEs platforms shall bring some breakthroughs to the prospect of future IEI treatment. These *in situ*, precise genome editing platforms provide unprecedented opportunities to “seamlessly” correct a faulty gene, while currently exhibiting good safety features. Taking the recent history of genome editing as a reference, it is safe to predict that many of the current technical burdens for precise genome editing of HSPCs would be removed in the foreseeable future. Novel platforms shall also arise in a rapid pace. Coupled with continued experiences from the ongoing IEI gene therapy trials (9), the future outlook is certainly bright for precise genome editing to become a powerful treatment option for more patients with these devastating conditions.

## Author contributions

QM, HS, and JL wrote and revised the manuscript. All authors contributed to the article and approved the submitted version.

## Funding

This work is supported by grants from the National Key Research and Development Program of China (2021YFF1000704 and 2019YFA0802802 to JL).

## Conflict of interest

The authors declare that the research was conducted in the absence of any commercial or financial relationships that could be construed as a potential conflict of interest.

## Publisher’s note

All claims expressed in this article are solely those of the authors and do not necessarily represent those of their affiliated organizations, or those of the publisher, the editors and the reviewers. Any product that may be evaluated in this article, or claim that may be made by its manufacturer, is not guaranteed or endorsed by the publisher.
